# Reduced forced vital capacity is independently associated with, aging, height and a poor socioeconomic status: a report from the Tunisian population-based BOLD study

**DOI:** 10.1186/s12890-022-02062-3

**Published:** 2022-07-11

**Authors:** Safa Hsan, Nadia Lakhdar, Imed Harrabi, Monia Zaouali, Peter Burney, Meriam Denguezli

**Affiliations:** 1 Laboratoire de recherche: Physiologie de l’exercice et physiopathologie: de l’intégré au moleculaire, LR19ES09, Faculté de Médecine de Sousse, Université de Sousse, 4000 Sousse, Tunisia; 2 Service d’épidémiologie, Faculté de Médecine de Sousse, Hôpital Farhat Hached, Université de Sousse, 4000 Sousse, Tunisia; 3 National Heart and Lung Institute, Imperial College London, London, UK; 4 Faculté de Médecine Dentaire de Monastir, Université de Monastir, Avenue Avicenne, Monastir, Tunisia

**Keywords:** Reduced forced vital capacity, Lung function, Spirometry, Risk factors, Prevalence

## Abstract

**Background:**

Reduced forced vital capacity (FVC) is a risk factor of all-cause mortality; however, the prevalence and determinants of reduced FVC are not available for the Tunisian population. This study investigated the association of reduced FVC with risk factors and health variables in an urban population of subjects aged ≥ 40 years and living in the city of Sousse in Tunisia.

**Methods:**

A cross-sectional survey was performed using data from the Tunisian Burden of Obstructive Lung Disease (BOLD) study. We defined reduced FVC as a post-bronchodilator FVC below the lower limit of normal using National Health and Nutrition Examination Survey (NHANES) values and Global Lung Function Initiative 2012 equations (GLI 2012) and determined the relation between this finding and the potential risk factors (demographic and socioeconomic factors and the presence of chronic diseases), using multivariable regression analysis.

**Results:**

The prevalence of reduced FVC was 26.6% (176/661) when using NHANES values for white Americans and 14.2% (94/661) using the GLI 2012 equations. Compared to people with normal FVC, those with a reduced FVC were significantly older, taller, had a lower body mass index (BMI), more respiratory symptoms and a higher prevalence of heart disease and hypertension. Multivariable analysis showed that reduced FVC was essentially driven by exposure to biomass smoke for heating, a number of schooling years lower than or equal to 6 years, a childhood history of hunger for a lack of money, aging and height.

**Conclusions:**

The prevalence of reduced FVC is associated with a poor socioeconomic status aging and height.

## Background

Accumulating findings indicate that reduced forced vital capacity (FVC) is significantly related to respiratory symptoms with various comorbid conditions, including cardiovascular diseases, metabolic syndrome, and obesity [1, 2]. Other studies have demonstrated a relation between survival and FVC [3] and have shown that reduced FVC may be a risk factor for higher mortality [3, 4] and can predict it better than systolic blood pressure or body mass index (BMI) [5].

The Atherosclerosis Risk in Communities (ARIC) study showed that African American individuals had a lower FVC than white individuals and that the higher mortality in African Americans could be explained by the lower FVC in this population [3]. Other studies showed a lower FVC in Africans compared to white individuals and explained it by differences in anthropometry, socioeconomic status, genetic background and environment between blacks and whites [6, 7]. In line with these findings, a high prevalence of reduced FVC has been found in a population of adults living in a West African city [8] and in asymptomatic Nigerians [9, 10].

However, in Tunisia, no studies are available on the prevalence and risk factors for reduced FVC. Because it may be a risk factor for higher mortality, reduced FVC deserves to be explored in the Tunisian population. Thus, in the present study, we aimed to evaluate the distribution of reduced FVC in a community-based study of a non-institutionalised sample of people aged more than 40 years living in the urban area of Sousse, using the Burden of Obstructive Lung Disease (BOLD) study methods. We also investigated potential risk factors leading to reduced FVC in the Tunisian population.

## Methods

We followed the BOLD protocol as it has been described elsewhere [11]. Ethics approval was obtained from the University Farhat Hached Hospital of Sousse ethics’ committee and all participants provided written informed consent before taking part.

### Study population

The survey was conducted on a representative gender-stratified random sample of non-institutionalized residents selected from the general population and living in Sousse (Tunisia). Home visits were scheduled for 807 adults aged ≥ 40 years to complete questionnaires and perform pre- and post-bronchodilator (BD) spirometry. Out of 730 eligible participants, 717 were eligible for spirometry and 661 completed the full protocol and met the quality control criteria. The exclusion criteria were mental illness, institutionalization, inability to conduct spirometry, and contraindications to salbutamol [11].

### Spirometry and bronchodilator reversibility test

We measured ventilatory function based on the joint American Thoracic Society (ATS)/European Respiratory Society (ERS) guidelines criteria using the hand-held EasyOne spirometer (ndd Medizintechnik AG, Zurich, Switzerland). Participants were tested by trained and certified technicians before and 15 min after administering two puffs of Salbutamol (200 μg) (Ventolin; GlaxoSmithKline, Middlesex, UK) [11]. Spirometers were daily calibrated, using a 3.00 L syringe and all spirograms were checked locally and then sent via a secure Internet transfer to the BOLD Study operations centre located at Imperial College, London, UK, for further quality control checks [12].

Acceptable spirograms had to consist of at least three trials, with two free from zero-flow errors, artifacts, termination prior to 6 s or before a plateau was evident on the volume-time curve, extra breaths, or coughing in the first second of the trial. For repeatability criteria, we accepted slightly greater variation in the highest and next-highest spirograms, if the difference was less than 200 ml, a slight relaxation of the ATS/ERS recommendation shown to have very little impact on quality while reducing missing data [13]. Analysis included only spirometry tests meeting these criteria.

### Questionnaire data

Questionnaire data were collected through face-to-face interviews administered by trained and certified staff in the participants’ native language. The standardised BOLD questionnaires were translated from English into Arabic and then back translated to ensure validity.

All participants completed a core questionnaire that included information on sociodemographic characteristics, respiratory diagnoses, disease symptoms, comorbidities, activity limitation and risk factors for respiratory diseases. Participants were also expected to complete an occupational questionnaire and (for current cigarette smokers) a “stages of change” questionnaire that assessed readiness to quit smoking. There was also a questionnaire to assess exposure to biomass fuels used at home for either heating or cooking.

### Definitions

FVC was considered reduced if it was less than the lower limit of normal (LLN) based on reference equations from participants in the Third National Health and Nutrition Examination Survey (NHANES) and in the Global Lung Function Initiative (GLI) 2012 using the GLI-2012 desktop software for large data sets (published on www.lungfunction.org) [14]. The diagnosis of COPD was considered in any patient who had a post-bronchodilator FEV_1_/FVC < LLN accompanied by symptoms of cough, sputum production, or dyspnea. COPD stages of severity were categorized according to GOLD guidelines (Stage I: if FEV_1_ ≥ 80% predicted; Stage II: if FEV_1_ ≥ 50 and < 80% predicted; Stage III: if FEV_1_ ≥ 30 and < 50%; and Stage IV: if FEV_1_ < 30% predicted) and using the proposed classification based on z-score of post-bronchodilator FEV_1_ (mild (z-score ≥  − 2), moderate (− 3 ≤ z-score <  − 2), severe (− 4 ≤ z-score <  − 3) and very severe (z-score <  − 4)).

Response rate was defined as the proportion of eligible respondents who completed both the post-bronchodilator spirometry (regardless of the quality) and the core questionnaire.

### Statistical analysis

All statistical tests were performed with IBM SPSS statistics software (version 23). The Kolmogorov–Smirnov test was used to test the continuous measures for normality. Normally distributed data were expressed as mean and standard deviation and were compared using an unpaired t-test and not normally distributed data were expressed as median and interquartile range (IQR) and compared using Mann Whitney U-test. Qualitative variables were compared using Chi squared or Fisher exact test. Calculations of odd ratios (ORs) and 95% CI values for reduced FVC in relation to potential risk factors were performed with multivariable logistic regression models. The predictors of reduced FVC that are associated with the greatest increase in R-squared value were introduced in the regression model. The final model included age category (40–49, 50–59, 60–69 and 70 + years), height, BMI, gender, smoking status, hunger for lack of money in childhood, education, occupational exposure to dusts/gases/fumes and exposure to biomass smoke for heating and to passive smoking during childhood. *P* value of < 0.05 was considered statistically significant.

## Results

### Sample description

Of a total sample of 807 subjects, 717 responders were interviewed, completed the core questionnaire and undertook spirometry (90% response rate). Among them, 661performed acceptable and reproducible post-BD spirometry.

No significant differences in age, sex and smoking status were found between responders and non-responders and the distribution pattern of these variables was similar in the two groups indicating that the study sample is highly representative of the general population.

Overall, the sample comprised subjects with secondary or higher level education (73.7%) who were overweight or obese (76.8%) and who were exposed to indoor air pollutants (50%).

40% of the participants gave a history of smoking, which was five times more prevalent in males than females. Among the smokers, 67% were current smokers.

About a quarter of the study subjects reported at least one of the three symptoms—cough, phlegm and wheeze in the past 12 months, however, only 9% of them reported a doctor diagnosed respiratory disease.

The most prevalent co-morbidities were hypertension (21%) and diabetes (11%). Gender differences in health status were noted in cough (*p* = 0.019), phlegm (*p* = 0.001) and doctor diagnosed respiratory diseases (*p* = 0.008). In each case, the rates were higher in men than in women.

### Prevalence and clinical characteristics of subjects with reduced FVC

Overall, the prevalence of reduced FVC was 25.9% for men (80/309) and 27.3% for women (96/352) when using the NHANES values for white Americans and 14.6% for men (45/309) and 13.9% for women (49/352) when using the GLI 2012 reference equation.

Subjects with reduced FVC were more likely to be older (*p* = 0.017), taller (*p* = 0.000) and to have a lower BMI (*p* = 0.007) compared to those with normal FVC (Table 1). However, there was no significant difference between the two groups in the number of years of schooling, smoking status and history of exposure to passive smoking during childhood, biomass smoke and occupational dusts/gases/fumes (Table 1).

**Table 1:** Comparison of characteristics of Sousse BOLD study participants with or without a reduced FVC^a^ using NHANES values

Characteristics	Overall participants (n = 661, 100%)		
	FVC > LLN (n = 485, 73.4%)	FVC < LLN (n = 176, 26.6%)	*p* value
Age, years, median (IQR)	52 (45–58)	53 (48–60)	**0.017**
Age group, years, n (%)			0.057
40–49	197 (40.6)	52 (29.5)	
50–59	192 (39.6)	79 (44.9)	
60–69	70 (14.4)	35 (19.9)	
≥ 70	26 (5.4)	10 (5.7)	
Male gender, n (%)	229 (47.2)	80 (45.5)	0.725
Height, m, median (IQR)	1.62 (1.55–1.70)	1.66 (1.59–1.72)	**< 0.001**
Weight, Kg, median (IQR)	77.1 (68.6–86.2)	76.3 (67.1–85.5)	0.600
BMI, Kg/m^2^, median (IQR)	29.0 (25.6–33.1)	27.4 (24.7–31.8)	**0.007**
Waist circumference, cm, median (IQR)	100.7 (91.0–109.0)	97.2 (89.4–106.5)	0.124
Hip circumference, cm, mean ± SD	103.9 ± 13.7	103.4 ± 11.9	0.664
Waist-hip ratio, mean ± SD	0.95 ± 0.09	0.94 ± 0.06	0.159
Abnormal waist circumference^b^, n (%)			
Male	99 (43.2)	33 (41.3)	0.758
Female	212 (82.8)	73 (76.0)	0.150
Abnormal waist-hip ratio^c^, n (%)			
Male	178 (77.7)	61 (76.3)	0.786
Female	243 (94.9)	91 (94.8)	1.000
History of hunger for lack of money at the age of five, n (%)	205 (42.3)	63 (35.8)	0.152
Education, yr of schooling, n (%)			**0.011**
7 +	275 (56.7)	80 (45.5)	
0–6	210 (43.3)	96 (54.5)	
Smoking status, n (%)			0.184
Current smoker	136 (28.0)	40 (22.7)	
Ex-smoker	58 (12.0)	29 (16.5)	
Never-smoker	291 (60.0)	107 (60.8)	
PB spirometric parameters, median (IQR)			
FEV_1_, L	2.84 (2.36–3.49)	2.07 (1.75–2.55)	**< 0.001**
FEV_1_, % predicted	97.16 (89.38–105.46)	76.53 (68.80–82.20)	**< 0.001**
FVC, L	3.53 (2.89–4.33)	2.61 (2.27–3.20)	**< 0.001**
FVC, % predicted	93.10 (86.80–100.64)	74.64 (69.85–78.67)	**< 0.001**
FEV_1_/FVC ratio, %	81.47 (77.31–85.20)	80.53 (75.88–84.27)	**0.023**
Respiratory symptoms, n (%)			
Chronic cough	44 (9.0)	31 (17.6)	**0.007**
Chronic wheeze	108 (22.2)	57 (32.4)	**0.008**
Chronic phlegm	72 (14.8)	30 (17.0)	0.488
Dyspnea	258 (53.1)	108 (61.7)	**0.049**
Reported diagnosis, n (%)			
Chronic bronchitis, emphysema or COPD	4 (0.8)	6 (3.4)	**0.026**
Hypertension	86 (17.7)	52 (29.5)	**0.001**
Diabetes	49 (10.1)	23 (13.1)	0.279
Heart disease	20 (4.1)	17 (9.7)	**0.006**
Stroke	1 (0.2)	0 (0.0)	1.000
Hospitalised as a child for breathing problems prior age 10	11 (2.3)	7 (4.0)	0.404
Exposure history, n (%)			
Exposure to passive smoking during childhood	306 (63.1)	99 (56.3)	0.110
Exposure to biomass smoke for heating^£^			**0.034**
Exposed	21 (4.3)	17 (9.7)	
Not exposed	221 (45.6)	75 (42.6)	
Never heat the home	243 (50.1)	84 (47.7)	
Exposure to biomass smoke for heating duration, years, median (IQR)	15.5 (10–30)	20 (10–30)	0.383
Exposure to biomass for cooking*	205 (42.3)	71 (40.3)	0.721
Exposure to biomass smoke for cooking duration, years, median (IQR)	10 (5.75–20.0)	15 (8.0–22.5)	0.092
Poor indoor ventilation	417 (86.0)	153 (86.9)	0.432
Dusty job^§^	108 (22.3)	36 (20.5)	0.618

Bold indicates burden of obstructive lung disease

*LLN* Lower limit of normal, *IQR* Interquartile range, *SD* Standard deviation, *FEV*_*1*_ Forced expiratory volume in 1 s, *FVC* Forced vital capacity, *BMI* Body mass index, *PB* Post bronchodilator

^a^FVC < LLN

^b^Abnormal waist circumference ≥ 102 cm for males and ≥ 88 cm for females

^c^Abnormal waist-hip ratio ≥ 0.90 for males and ≥ 0.85 for females

^£^Exposure to indoor open fire with wood, coal or drop residue as a primary means of heating home for at least 6 months

^*^Exposure to to indoor open fire with wood, coal or drop residue as a primary means of cooking for at least 6 months

^§^Ever worked in a dusty job for at least 1 year

Unlike those with normal FVC, subjects with a reduced FVC were more likely to have chronic cough, chronic wheeze and dyspnoea (Table 1). Moreover, reported diagnosis of chronic bronchitis, emphysema or COPD was significantly more frequent among subjects with reduced FVC versus those with normal FVC (*p* = 0.026).

Regarding comorbidities, hypertension and heart disease were significantly more prevalent in subjects with a reduced FVC compared to those with a normal FVC while there were no significant differences in the prevalence of diabetes and stroke between the two groups (Table 1).

Surprisingly, a large percentage of subjects with reduced FVC were never smokers (60.8%). This appears clearly especially in women with a reduced FVC since 89.6% of them had never smoked.

### Lung function parameters

In general, the analysis suggested that the subjects with a reduced FVC had poorer lung function than the subjects with normal FVC (Table 1). Deficits in FEV_1_, FVC and FEV_1_/FVC were found in both men and women, across the age range. However, women with reduced FVC have less chronic cough and produced less sputum than men but suffered more frequently from dyspnoea (*p* < 0.05).

### Risk factors for reduced FVC

We performed univariable and multivariable logistic regressions to assess the association of LLN-based reduced FVC with age, height, gender, BMI, smoking status, hunger for lack of money in childhood, education, occupational exposure to dusts/gases/fumes, and exposure to biomass smoke for heating and to passive smoking during childhood (Table 2). After mutual adjustment for all these potential factors in the model, we found that in our study population, the significant determinants of reduced FVC included, aging, height, a childhood history of hunger for a lack of money, having less than 7 years of schooling and a history of exposure to biomass smoke for heating.

**Table 2:** Factors associated with FVC less than lower limit of normal using NHANES equation

Variables	Categories	Univariate model			Multivariate model		
		OR	95% CI	*p* value	OR	95% CI	*p* value
Age group, yr	40–49 (reference)	1			1		
	50–59	1.559	1.042–2.331	**0.031**	1.469	0.982–2.289	0.076
	60–69	1.894	1.140–3.148	**0.014**	1.849	1.031–3.315	**0.039**
	≥ 70	1.457	0.661–3.213	0.351	1.312	0.551–3.120	0.540
Height, m		1.029	1.010–1.048	**0.002**	1.026	1.005–1.047	**0.014**
Body mass index, Kg/m^2^	BMI < 20	1.799	0.990–3.271	0.054	1.395	0.730–2.665	0.313
	20 ≤ BMI < 25 (reference)	1			1		
	25 ≤ BMI < 30	1.535	0.566–1.164	0.400	1.206	0.420–3.463	0.728
	30 ≤ BMI < 35	1.540	0.890–2.663	0.122	1.122	0.625–2.016	0.700
	BMI ≥ 35	1.017	0.559–1.848	0.957	0.831	0.447–1.545	0.558
Sex	Women	1.073	0.759–1.517	0.688	0.976	0.570–1.670	0.929
Smoking status	Non-smoker (reference)	1			1		
	Current smoker	1.700	0.963–3.001	0.067	1.459	0.796–2.677	0.222
	Former smoker	1.250	0.824–1.896	0.293	1.130	0.656–1.946	0.660
Ever go hungry for lack of money at the age of five	Yes	1.313	0.919–1.877	0.135	1.466	1.000–2.151	**0.050**
Education, yr of schooling	7 + (reference)	1			1		
	0–6	1.571	1.111–2.223	**0.011**	1.625	1.087–2.430	**0.018**
Dusty job^§^	Yes	0.856	0.553–1.326	0.487	1.193	0.705–2.016	0.511
	Don’t work	0.821	0.518–1.302	0.402	1.066	0.625–1.820	0.814
Passive smoking during childhood	Exposed	1.330	0.937–1.887	0.111	1.226	0.847–1.775	0.280
Exposure to biomass smoke for heating^£^	Never heat the home (reference)	1			1		
	Exposed	2.342	1.179–4.650	**0.015**	2.743	1.338–5.626	**0.006**
	Not exposed	0.982	0.684–1.408	0.920	1.032	0.711–1.500	0.867

Bold indicates burden of obstructive lung disease

*OR* Odds ratio, *CI* Confidence interval

^§^Ever worked in a dusty job for at least 1 year

^£^Exposure to indoor open fire with wood, coal or drop residue as a primary means of heating home for at least 6 months

## Discussion

In this population of urban residents of Sousse, Tunisia, we found that more than one in four adults had a reduced FVC, which was essentially driven by aging, height, a childhood history of hunger for a lack of money, having less than 7 years of schooling and a history of exposure to biomass smoke for heating.

Since reduced FVC is an important determinant of mortality risk in the general population [3, 15–17], our findings indicate the importance of using the identified determinants to select from the general population subjects who are more likely to be diagnosed with a reduced FVC.

According to Burney et al. findings in a general population cohort of asymptomatic adults without chronic respiratory diagnoses or persistent respiratory symptoms, survival is associated with FVC and not with airway obstruction as measured by the FEV_1_/FVC ratio and FEV_1_ [3].

The prevalence of reduced FVC pattern in our study was higher than the 26.4% reported by de la Hoz et al., [18] in the workers and volunteers exposed to dust, gases and fumes at the World Trade Center disaster site in 2001–2002 and diagnosed in 2018. Obaseki et al., found a prevalence of 70.4% for reduced FVC among adults aged 40 years and older participating in the BOLD study in Ile-Ife, Nigeria, that was far higher than the 26.6% found in our sample [8]. However, in the study of Cooksley et al. [19] comprising Aboriginal and non-Indigenous residents of the Kimberley region of Western Australia aged 40 years or older and following the BOLD protocol, the prevalence of reduced FVC defined by a FVC < 80% varied from 9.7% in non-Indigenous to 74.0% in Aboriginal participants.

Global variations in the prevalence of reduced FVC pattern can be explained by regional differences in the distribution of a number of factors including participants’ demographics, socio-economic status, prevalence and type of comorbidities and prevalence of restrictive lung diseases. However, that restrictive diseases are rather rare in the general population suggests that they contribute less to the burden of reduced FVC prevalence across published studies [20].

In our population and according to the multivariable analysis, increasing age constitutes a statistically significant risk factor of reduced FVC (OR = 1.849; 95% CI = 1.031, 3.315; *p* = 0.039) (Table 2). This finding is in line with previous studies results [21] and is relatively not surprising since age has historically been one of the major factors in the evaluation of lung function [22]. Indeed, pulmonary maturity peaks around the age of 20–25 years, after which lung function progressively begins an accelerated decline in FEV_1_, FVC and FEV_1_/FVC ratio and an increase in residual volume and functional reserve capacity [22]. The lung function decline is likely explained by the combined effect of several phenomena occurring with aging which include deformation of the axial skeleton, deterioration of the elasticity and recoil capacity of the lung tissues and lung aging.

The finding that reduced FVC prevalence increases with age does not minimize the fact that, in the present study, height constitutes a potential risk factor of reduced FVC (OR = 1.026; 95% CI = 1.005, 1.047; *p* = 0.014). An extensive bibliography is available on how anthropometric parameters affects lung morphology and function. FVC is affected by height, since it is proportional to body size [23]. This means that in tall individuals, who accordingly have greater lung capacity, lung volume will decrease at a greater rate to shorter individuals as they grow older [24, 25]. This process is favoured by smoking and / or exposure to air pollution such as exposure to biomass smoke, which constitutes an important risk factor for reduced FVC [26–28]. Indeed, our study demonstrated that exposure to indoor biomass is an influential risk factor of reduced FVC (OR = 2.743; 95% CI = 1.338, 5.626; *p* = 0.006). In the present study, 40.3% of the subjects with reduced FVC were exposed to biomass for cooking and 39.2% have been exposed to coal smoke for heating for 17 and 20 years, respectively. Moreover, 87% of our participants had poor indoor ventilation conditions and were then exposed to a more concentrated smoke. These results are consistent with the findings of other studies from developing countries, such as sub-Saharian African city, rural Nepal and Turkey showing elevated prevalence of respiratory symptoms and reduced lung function in association with solid fuel utilisation [26, 27, 29]. According to Balcan et al. [30], intensive exposure of household members to biomass fuel smoke and particles could accelerate the reduction of FVC especially in case of long-term exposition. Moreover, Cooksley et al., reported that, in Aboriginal Australians, exposure to domestic combustion could accelerate age-related loss of lung function and partially account for a reduced FVC [19].

The use of coal for heating and cooking is very widespread in Tunisia and is related to the poor socioeconomic status of the Tunisians. The participants to this study have certainly been exposed to biomass fumes since their childhood increasing, then, their exposure at the critical times of lung development. A reduction of FVC was also demonstrated in patients who were exposed to biomass smoke based on radio-imaging findings consistent with restrictive pathology and histologic problems, such as septal enlargements, metaplasia of goblet cells, glandular hyperplasia, and fibrosis [31, 32].

In line with these findings, a positive association was found between reduced FVC and a participants’ number of schooling years lower than or equal to 6 years (OR = 1.625; 95%CI = 1.087, 2.430; *p* = 0.018). There is a significant body of literature supporting these findings and showing that the level of education is a risk factor for reduced maximal ventilatory function and chronic lung disease in adults [33–35]. The association we found between reduced FVC and a childhood history of hunger for a lack of money (OR = 1.466; 95%CI = 1.000, 2.151; *p* = 0.050) supports these findings. Inequalities in lifelong respiratory health originate in childhood when adequate nutrition is essential [34, 36, 37]. Indeed, from conception until adolescence, the respiratory system develops rapidly especially during the first 3 years of life. Disruption to this development in childhood is one of the several consequences of living in poverty, including malnutrition [37]. These early-life disruptions influence anthropometry and constitute risk factors for a lung function decline [37]. We used this last factor and the education level as indicators of a poor socioeconomic status [37] a risk factor that encapsulates exposure to several agents affecting foetal lung development and postnatal lung growth [8]. The very high prevalence of reduced FVC seen in Sousse, Tunisia, is consistent with the low socioeconomic status of the Tunisians and had been previously reported by other BOLD Study sites with similar economies such as Nigeria [8], Malawi [38] and South Asia [39].

Interestingly, in the present study, a large percentage of subjects with reduced FVC were never smokers (60.8%). Moreover, 89.6% of the women with a reduced FVC had never smoked. In line with these findings, no association was found between a reduced FVC and smoking habits. These results support the role of risk factors, other than tobacco smoking, that may contribute to a reduced FVC and include childhood respiratory tract infection, low social status, nutrition and environmental pollution exposure [40].

Although cardiovascular diseases had been reported to be associated with a reduced FVC [41], this was not found in the present study. The cardiovascular diseases are known to be highly under-diagnosed and the information collected on the status of the disease in our sample was based on subjects’ self-report. It is very likely that our sample includes a high number of undiagnosed cardiovascular diseases and the latter could obliterate any real association.

A link between diabetes and reduced FVC has also been reported from many studies including ARIC [42], and has more often been ascribed to an adverse effect of diabetic pathology on the ventilatory function [43], a view that is supported by a more rapid decline of lung function in subjects with diabetes [44]. However, in our study, the correlation between diabetes and reduced FVC did not reach the statistical significance level.

## Conclusions

In a population-based study of adults living in a Tunisian city, we found a high prevalence of reduced FVC similar to the prevalence found in other sites where the economic conditions are similarly poor. Reduced FVC was independently associated with exposure to biomass fumes for heating, a number of schooling years lower than or equal to 6 years, a childhood history of hunger, aging and height,. These findings support the view that the potential contribution of environmental factors to reduced FVC should be considered rather than assuming that having a small lung volume is accounted for solely by genetic factors. Our findings also indicate the importance of using the identified risk factors to select from the general population, subjects who are more likely to be diagnosed with reduced FVC, and to benefit from targeted risk factors control to mitigate the adverse outcomes.

## Data Availability

The datasets used and/or analysed during the current study are available from the corresponding author on reasonable request.

## Abbreviations

ATS: American thoracic society
BD: Bronchodilator
BMI: Body mass index
BOLD: Burden of obstructive lung disease
CI: Confidence interval
COPD: Chronic obstructive pulmonary disease
ERS: European respiratory society
FEV_1_: Forced expiratory volume in 1 s
FVC: Forced vital capacity
GLI 2012: Global lung function initiative 2012
IQR: Interquartile range
LLN: Lower limit of normal
NHANES: National Health and Nutrition Examination Survey
OR: Odds ratio
SD: Standard deviation
